# An updated history of TDIF‐PXY signalling: a study in cell fate and tissue patterning

**DOI:** 10.1111/nph.70508

**Published:** 2025-08-29

**Authors:** Qing He, Agnieszka Gladala‐Kostarz, Elizabeth E. Morris, J. Peter Etchells

**Affiliations:** ^1^ Department of Biosciences Durham University Stockton Road Durham DH1 3LE UK

**Keywords:** cambium, development, meristem, phloem, signalling, vasculature, xylem

## Abstract

Non‐cell autonomous signalling is a mechanism by which stem cells are maintained across the tree of life. In plants, many stem cell populations are regulated by peptide ligands related to CLAVATA3, which are secreted from one cell type and bind to the leucine‐rich repeat domain of a plasma‐membrane‐localised receptor kinase related to CLAVATA1 in an adjacent cell type. Activation of CLAVATA1‐like receptors then leads to a suite of events regulating stem cell fate. The vascular cambium, the meristem from which xylem and phloem are derived, is no different in this respect. Here, TRACHEARY ELEMENT DIFFERENTIATION FACTOR (TDIF) peptide is excreted from phloem cells and binds to the PHLOEM INTERCALATED WITH XYLEM (PXY) receptor kinase, which in turn leads to activation of a suite of factors that influence xylem and phloem production. In 2016, we reviewed the literature that described signalling components, phytohormones and transcription factors that interacted with TDIF and PXY. In the intervening period, our understanding of these interactions has significantly progressed and here we provide an update, describing how TDIF and PXY promote growth while maintaining pattern in the stem cell populations that they regulate.

**Table:** 

	Contents	
	Summary	1154
I.	Introduction	1155
II.	The TDIF‐PXY ligand‐receptor pair represents an ancient innovation	1155
III.	The structural basis of TDIF perception by PXY	1157
IV.	Events that occur upon binding of TDIF by PXY (i): WOX14‐TMO6‐LBD4 feed‐forward loop regulation	1158
V.	Events that occur upon binding of TDIF by PXY (ii): regulation of MONOPTEROS	1159
VI.	Events that occur upon binding of TDIF by PXY (iii): regulation of AIL expression to define the cambium stem cells	1160
VII.	Parallel pathways to TDIF‐PXY (i): the ERECTA family	1161
VIII.	Parallel pathways to TDIF‐PXY (ii): ethylene	1162
IX.	Parallel pathways to TDIF‐PXY (iii): cytokinin	1162
X.	Perspectives	1163
	Acknowledgements	1163
	References	1163

## I. Introduction

A prerequisite to perpetual growth in plants is the maintenance of discrete stem cell populations. In plants, such stem cells are maintained in specialized tissues known as meristems. Here, cell division and the growth derived from those cell divisions occur continuously, supporting plants' lifelong growth and developmental plasticity (Dinneny & Benfey, 2008; Hirakawa *et al*., 2010). Two indeterminate meristems, the root apical meristem and shoot apical meristem, are established during embryogenesis, which contributes to the formation of primary structures; the root apical meristem gives rise to the root system, the shoot apical meristem is the stem cell population from which all aerial tissues including leaves and flowers are derived, and the procambium hold the stem cells from which vascular tissue forms in primary growth (Taiz & Zeiger, 2002). Secondary growth, responsible for the thickening of the plant, is controlled by the two lateral meristems, the cork cambium and vascular cambium. These meristems are defined following germination. The cork cambium is involved in the production of periderm on the outer surface of the plant for protective purposes. The vascular cambium is typically located between the xylem and phloem in the stems and roots of woody gymnosperms and eudicot angiosperms. The vascular cambium forms a continuous cylinder and is the main driver of secondary growth. This occurs via mostly periclinal cell divisions parallel to the closest organ surface. Cambium is bifacial in nature, with xylem derived from cambial stem cell divisions towards the inside of the stem, while phloem forms from cell divisions towards the outside (Barlow *et al*., 2002; Bossinger & Spokevicius, 2018; Smetana *et al*., 2019).

Many studies have described molecular and genetic components that regulate cambium and procambium activity to facilitate the development of plant vascular tissues, including phytohormones, transcription factors and signalling mechanisms (Immanen *et al*., 2016; Fischer *et al*., 2019; Hunziker & Greb, 2024). Among these, the TDIF‐PXY ligand‐receptor pair has emerged as a central regulator. TRACHEARY ELEMENT DIFFERENTIATION FACTOR (TDIF) is a phloem‐derived dodecapeptide ligand, derived from *CLAVATA3/ESR RELATED 41* (*CLE41*) and *CLE44*, which is perceived by the PHLOEM INTERCALATED WITH XYLEM (PXY) receptor kinase present in the cambium. PXY is constituted of an extracellular ligand‐binding domain of 22 leucine‐rich repeats (LRRs), a single‐helix transmembrane domain and a cytoplasmic kinase domain which is activated upon TDIF perception. In *Arabidopsis thaliana*, PXY is held to act redundantly with closely related receptor kinases PXY‐LIKE 1 (PXL1) and PXL2. Early discoveries, reviewed in Etchells *et al*. (2016), also included identification of transcriptional‐target transcription factors WUSCHEL‐RELATED HOMEOBOX 4 (WOX4) and WOX14 which promote vascular cell division; crosstalk between PXY and ethylene signalling; interactions between the PXY kinase domain and GSK3 kinases that resulted in degradation of BES1, a transcription factor that promotes xylem differentiation; and the role of TDIF‐PXY in lateral root initiation.

However, since 2016, our understanding of TDIF‐PXY signalling has improved significantly; thus, the aim of this review article is to place these new discoveries in context. The principal players are summarised in Table 1. Advances in our understanding include insights from the structure of the TDIF‐PXY complex, identification of new factors that TDIF‐PXY regulates and with which it cross‐talks, and a better understanding of how TDIF‐PXY function arose and was modified in the green plant lineage.

**Table 1:** Glossary of *Arabidopsis thaliana* genes in this review.

Abbreviation	Gene name	Molecular function	Summary of function
ACS	1‐aminocyclopropane‐1‐carboxylate synthase	Enzyme	Key enzyme in the biosynthesis of ethylene
AHK	ARABIDOPSIS HISTIDINE KINASE	Receptor kinase	Regulates cytokinin signalling to control plant growth and organ development
AHL	ARABIDOPSIS HISTIDINE KINASE‐LIKE	Transcription factor	Regulates gene expression in response to cytokinin signalling, influencing growth
ANT	AINTEGUMENTA	Transcription factor	Controls meristematic activity
ARF	AUXIN RESPONSE FACTOR	Transcription factor	Mediates the response to auxin
ARR	ARABIDOPSIS RESPONSE REGULATOR	Transcription factor	Mediates cytokinin response
BAK1	BRI1‐ASSOCIATED RECEPTOR KINASE 1	Receptor kinase	Co‐receptor in several ligand‐receptor signalling mechanisms
BES1	BRI1‐EMS‐SUPPRESSOR 1	Transcription factor	Promotes vascular tissue differentiation in response to brassinosteroid signalling
BIL	BRI1‐ASSOCIATED RECEPTOR KINASE	GSK3‐like kinase	Affects vascular tissue patterning and differentiation
BIN2	BRASSINOSTEROID INSENSITIVE 2	GSK3‐like kinase	Inhibits vascular tissue differentiation via brassinosteroid signalling repression
BRI1	BRASSINOSTEROID INSENSITIVE 1	Receptor kinase	Brassinosteroid receptor
CKI1	Cytokinin‐Inducible 1	Kinase	Mediates cytokinin responses that influence cell division and vascular development
CLE41/44	CLAVATA3/EMBRYO SURROUNDING REGION 41/44	TDIF peptide ligand precursor	Regulates vascular meristem activity
CLV1	CLAVATA1	Receptor kinase	CLV3 receptor controlling shoot apical meristem size
CLV3	CLAVATA3	Peptide ligand	Ligand to the CLV1 receptor kinase
EPFL	EPIDERMAL PATTERNING FACTOR‐LIKE	Peptide ligand	Ligand to the ER receptor kinase
ER	ERECTA	Receptor kinase	Regulates multiple aspects of plant development including vascular development
ERF	ETHYLENE RESPONSE FACTOR	Transcription factor	Modulates gene expression in response to ethylene and environmental stresses
ERL	ERECTA‐LIKE	Receptor kinase	Acts redundantly with ER in some aspects of plant development
FLS2	FLAGELLIN SENSITIVE 2	Receptor kinase	Involved in immune response
GSK3	Glycogen Synthase Kinase 3	Protein kinase	Controls cell proliferation and differentiation
HD‐ZIP III	HOMEODOMAIN‐LEUCINE ZIPPER III	Transcription factor	Regulates xylem differentiation and adaxial identity
IPT	Isopentenyltransferase	Enzyme	Synthesizes cytokinins
LBD4	LATERAL ORGAN BOUNDARIES DOMAIN 4	Transcription factor	Regulates vascular development
LOG4	Longevity‐Associated Gene	Biosynthesis gene	Catalyzes cytokinin biosynthesis
MP	MONOPTEROS	Transcription factor	Regulates auxin‐mediated patterning of vascular tissues
PLT/AIL	PLETHORA/ AINTEGUMENTA‐like	Transcription factor	Regulates stem cell populations
RhPMP1	*PETAL MOVEMENT‐RELATED PROTEIN1*	Transcription factor	Regulated by ethylene
PXL1	PXY‐LIKE 1	Receptor kinase	Functions redundantly with PXY to regulate vascular meristems
PXL2	PXY‐LIKE 2	Receptor kinase	Functions redundantly with PXY to regulate vascular meristems
PXY/TDR	PHLOEM INTERCALATED WITH XYLEM/TDIF RECEPTOR	Receptor kinase	Regulates vascular meristem development
SERK	SOMATIC EMBRYOGENESIS RECEPTOR KINASE	Receptor kinase	Co‐receptor to several receptor kinases
SOBIR1/EVR	SUPPRESSOR OF BIR1/EVERSHED	Receptor kinase	Regulates xylem fibre development
TDIF	TRACHEARY ELEMENT DIFFERENTIATION FACTOR	Peptide ligand	PXY ligand
TMM	TOO MANY MOUTHS	Receptor protein	Forms receptor complex with ER
TMO6	TARGET OF MONOPTEROS 6	Transcription factor	Regulates vascular development in response to cytokinin signalling
WOX4	WUSCHEL‐RELATED HOMEOBOX 4	Transcription factor	Promotes cambium proliferation in response to TDIF signalling
WOX14	WUSCHEL‐RELATED HOMEOBOX 14	Transcription factor	Works with WOX4 to sustain cambium proliferation and vascular development
XVP	PRECOCIOUS XYLEM DIFFERENTIATION AND ALTERED VASCULAR PATTERNING	Transcription factor	Regulates vascular tissue differentiation

## II. The TDIF‐PXY ligand‐receptor pair represents an ancient innovation

Non‐cell autonomous signalling represents a mechanism by which cell division and differentiation are controlled in many stem cell systems. TDIF‐PXY was first discovered as a regulator of vascular development, raising the question of where this innovation arose in the tree of life; that is, whether it arose after the evolution of vascular plants as a mechanism to modulate vascular development, concomitant with the evolution of vascular tissue in plants, or before the evolution of vascular plants.

Across the bryophytes, comparative genomic and phylogenetic analyses reveal a conserved presence of single TDIF‐like genes in both liverworts and hornworts, including in *Marchantia polymorpha* and *Anthoceros agrestis*. This suggests that the ancestral land plant genome contained TDIF (Whitewoods *et al*., 2018; Frangedakis *et al*., 2021; Furumizu & Shinohara, 2024). The homologues of TDIF and PXY in *Marchantia polymorpha* are *MpCLE1* and *MpTDR*. Analysis revealed that the MpCLE1 peptide suppresses the growth of the *Marchantia polymorpha* thallus and lines overexpressing *MpCLE1* produced fewer gemmae cups and did not form gametangiophores. Furthermore, *MpCLE1* reduces proliferative activity in the apical notch, which distorts thallus growth. Expression analysis showed that *MpCLE1* and its receptor gene *MpTDR* are expressed in distinct patterns across the apical notch region, with *MpCLE1* expressed in ventral regions and *MpTDR* demonstrating broader expression along the dorsoventral axis (Hirakawa *et al*., 2019; Takahashi *et al*., 2023).

By contrast, mosses such as *Physcomitrium patens* show no evidence of having TDIF‐like CLE genes, based on genome searches and gene tree analyses, suggesting loss of these genes (Whitewoods *et al*., 2018); although nine other CLE family genes are retained (Cammarata *et al*., 2022; Nemec‐Venza *et al*., 2022, 2025; Shumbusho *et al*., 2024). Two of these, PpCLE1 and PpCLE2, are functionally homologous to the Arabidopsis CLV3 peptide, which regulates apical meristems in angiosperms (Fletcher *et al*., 1999), rather than to TDIF. These genes regulate cell proliferation and gametophore apex function in *Physcomitrium* (Whitewoods *et al*., 2018).

While CLE peptide signalling components, including TDIF and PXY‐like receptors, are found in some species within the bryophyte lineage, as they lack true vascular tissues, the suggestion is that the canonical TDIF‐PXY system likely emerged later in evolution alongside the development of vascular cambium in tracheophytes (Hirakawa *et al*., 2019; Takahashi *et al*., 2023). Indeed, evolutionary analyses indicate that PXY‐like receptors and TDIF‐type CLE peptides are present in lycophytes, ferns, gymnosperms and angiosperms, suggesting that the core components of this signalling system arose in the last common ancestor of vascular plants (Hirakawa & Bowman, 2015; Whitewoods *et al*., 2018; Renninger *et al*., 2025). To test whether TDIF activity was conserved among vascular plants, Hirakawa & Bowman (2015) performed comparative analyses of TDIF signalling in non‐flowering vascular plants (gymnosperms, ferns and lycophytes). They identified orthologs of TDIF and PXY in *Ginkgo biloba* (gymnosperm), *Adiantum aethiopicum* (fern) and *Selaginella kraussiana* (lycophyte) by RACE‐PCR. Exogenous TDIF application in *Ginkgo biloba* and *Adiantum aethiopicum* suppressed xylem differentiation in procambial cells of leaves. However, TDIF treatment did not affect developing shoots and rhizophores of *Selaginella kraussiana* (Hirakawa & Bowman, 2015). One explanation for this observation is that the role of TDIF‐PXY in vascular development is limited to ferns and seed plants.

TDIF‐PXY signalling is best characterised in its central role in regulating vascular development in the eudicot lineage of flowering plants. This function is conserved in plants with dramatically different growth strategies, such as rapid cycling annuals like Arabidopsis (Ito *et al*., 2006; Fisher & Turner, 2007; Hirakawa *et al*., 2008; Etchells & Turner, 2010) and woody perennials such as Populus (Etchells *et al*., 2015; Kucukoglu, 2017). Less is known about TDIF‐PXY function in monocot species; however, studies in wheat and switchgrass suggest that TDIF‐PXY signalling is conserved. It remains an open question as to whether its role may be functionally divergent due to, for example, evolutionary modifications to suit monocot vascular architecture, which, unlike that of eudicots, is not a driver of secondary growth. Evolutionary analysis in wheat revealed that only one protein (TaCLE29) has the same mature peptide sequence as TDIF peptides in Arabidopsis. Expression of TaCLE29 is predicted to be high in all wheat tissues. Moreover, using phylogenetic analysis, two TaCLE29 protein homologues were identified in rice (Li *et al*., 2019).

In switchgrass (*Panicum virgatum*), five TDIF‐like CLE genes (*PvTDIFL1‐PvTDIFL5*) have been identified. Particularly, PvTDIFL1 and PvTDIFL3 variants showed enriched expression in the rachis. Functional assays in Arabidopsis demonstrated that exogenous application of PvTDIFL peptides induced increased stele cell proliferation, similar to the effects of endogenous TDIF peptides. Moreover, heterologous expression of *PvTDIFL1* in Arabidopsis resulted in inhibited growth, enhanced vascular cell division and disrupted cellular organisation of the hypocotyl. This suggests that the function of PvTDIFLs is similar to Arabidopsis TDIF (Tian *et al*., 2021). Given the distinct anatomical features of monocot stems relative to those of dicots, a question ripe for further research concerns evolutionary divergence in how TDIF‐PXY signalling functions in these different angiosperm clades.

## III. The structural basis of TDIF perception by PXY

**Fig. 1:**
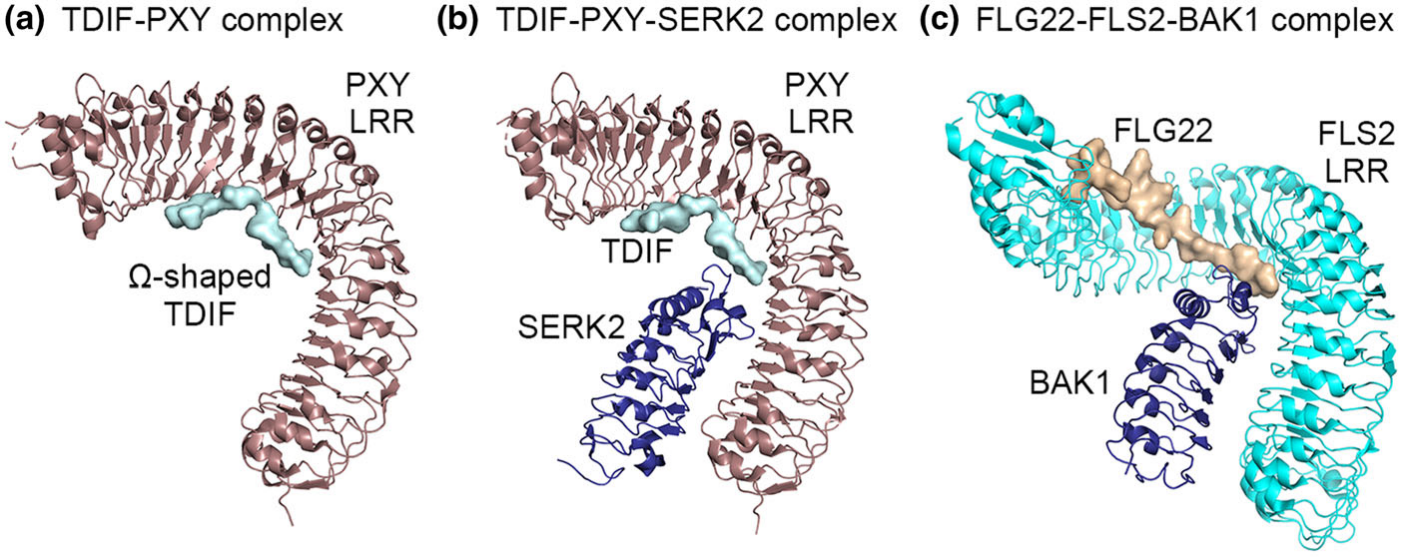
Comparison of *Arabidopsis thaliana* PHLOEM INTERCALATED WITH XYLEM (PXY) and FLS ligand‐receptor complexes. (a) Crystal structure of TDIF‐PXY complex showing Ω‐shaped TDIF in the binding pocket of the LRR domain of PXY. (b) Crystal structure of TDIF‐PXY‐SERK2 complex. Changes to structure are negligible in the present or absence of SERK2. (c) Crystal structure of FLG22 in complex with FLS2 and BAK1. The FLS2 ectodomain has a greater degree of twist than PXY. Panels (a) and (b) were generated from structures described in Zhang *et al*. (2016a, 2016b); panel (c) was generated from a structure described in Sun *et al*. (2013b).

Across the broad range of plant species carrying TDIF and PXY homologues, activation of PXY by TDIF is dependent upon the presence of TDIF within the binding pocket of the PXY extracellular LRR domain. In PXY, the extracellular domain forms a twisted right‐handed superhelix with 22 leucine‐rich repeats (LRRs), N‐terminal (residues 34–81) and C‐terminal (residues 609–637) capping domains (Morita *et al*., 2016; Zhang *et al*., 2016a). Structural analysis revealed that TDIF primarily adopts an ‘Ω’‐like conformation as it binds to the inner concave surface of the LRR domain of PXY (Fig. 1a). The interaction between TDIF and PXY is mainly facilitated by conserved amino acids in TDIF. Key conserved residues in TDIF include hydroxylated proline (Hyp) residues, a C‐terminal asparagine (Asn) and a central glycine (Gly) (Zhang *et al*., 2016a). The canonical TDIF peptides contain two hydroxyproline (Hyp) residues‐Hyp4 and Hyp7‐that are essential for PXY recognition. These Hyp residues form hydrogen bonds with the LRR domain of PXY. Hyp7 is particularly crucial, as mutations, such as Hyp → Ala, disrupt binding. The C‐terminal Asn stabilizes the peptide‐receptor interaction via polar contacts and mutations at that position, such as Asn → Ala, reduce binding affinity. The central Gly provides structural flexibility, enabling proper docking into the PXY binding pocket. These two residues, the C‐terminal Asn and central Gly at position 6 in TDIF, are highly conserved among CLE peptides, as is the valine at position 3. Structure‐based sequence alignment has suggested that the conserved TDIF‐interacting motifs are predominant in other known CLE/LRR receptor kinase interactions (Zhang *et al*., 2016a; Li *et al*., 2017).

Comparisons of the TDIF‐PXY complex structure with those of other plant ligand‐receptor pairs revealed commonalities and differences. Differences in the arrangement of receptor, co‐receptor and ligand likely lie in the number of leucine rich repeats (LRRs). For example, receptor kinases BRASSINOSTEROID INSENSITIVE 1 (BRI1) which regulates growth and differentiation via brassinosteroid perception, have 25 LRR repeats and FLAGELLIN SENSITIVE 2 (FLS2) which regulates immune responses, has 28; both greater than the 22 in PXY. The degree of twist in the LRR domain also differs, with PXY demonstrating less than that observed in other LRR receptor kinases (LRR‐RK), such as FLS2 among others, providing a flatter surface of ligand interaction (Zhang *et al*., 2016a; Sun *et al*., 2013b; Fig. 1).

Many ligand‐LRR‐RK pairs require a co‐receptor to facilitate ligand binding, with the co‐receptor forming part of the binding pocket. For example, both BRI1 and FLS2 require members of the SOMATIC EMBRYOGENESIS RECEPTOR KINASEs (SERKs) as co‐receptors. Here, both RK and SERK contact with the ligand to form a SERK‐LRR‐RK complex. SERK‐receptor pairs form via non‐specific van der Waals contacts between the concave surface of a SERK co‐receptor and the lateral side of the receptor kinase. The non‐specific nature of the interaction explains why SERKs interact with so many LRR‐RLKs. A conserved loop on SERKs also interacts with the LRR domain of its co‐receptor, which is essential for the formation of the SERK‐ligand‐receptor kinase ternary complex (Santiago *et al*., 2013; Sun *et al*., 2013a). Evidence to suggest that SERKs are important for PXY‐mediated signal transduction comes from a *serk1 bak1 serk2* triple mutant, where plants had a *pxy*‐like phenotype (*BAK1* being a member of the SERK family). Furthermore, a NAC transcription factor XVP interacts with BAK1 at the plasma membrane and modulates PXY signalling (J.H. Yang *et al*., 2020), providing further evidence of the involvement of SERKs.

Using gel‐filtration analysis performed at an acidic pH, it was shown that the extracellular LRR domain protein of SERK2 (SERK2^LRR^) formed a stable complex with the extracellular LRR domain protein of PXL2 (PXL2^LRR^) in the presence of the peptide derived from CLE42 (Mou *et al*., 2017), which has a single amino acid change relative to TDIF but similar activity (Ito, 2006). SERKs have also been shown to interact with PXY, as the crystal structure of PXY‐TDIF‐SERK2 complexes shows that SERK2 binds to the concave side of the C terminus of PXY and forms bonds with TDIF (Fig. 1b). This binding does not induce any obvious large structural rearrangement of PXY, as demonstrated by the closely matched structure between the PXY‐TDIF pair and the PXY‐TDIF‐SERK2 ternary complex (Zhang *et al*., 2016b). PXY^LRR^ interacts with SERK2^LRR^ mainly through van der Waals contacts with SERK2 Phe64, making extensive interactions with LRR16‐18 of PXY. In addition to the direct interactions between SERK2^LRR^ and PXY^LRR^, the interaction could also be mediated by TDIF, with its C‐terminal side being sandwiched between SERK2^LRR^ and PXY^LRR^, similar to the flg22‐mediated FLS2‐BAK1 interaction (Sun *et al*., 2013b; Zhang *et al*., 2016b).

Given that the TDIF‐PXY structure demonstrates negligible structural change in the presence or absence of a SERK co‐receptor, at least part of the response to TDIF‐PXY binding may be independent of SERK co‐receptors. This is supported by the observation that when the co‐receptors were mutated, these *serk1 bak1 serk2* plants demonstrated similar levels of *WOX4* expression as wild‐type controls upon treatment with TDIF (Zhang *et al*., 2016b), *WOX4* being a transcriptional target of TDIF‐PXY signalling (Hirakawa *et al*., 2010). This suggests that the SERK co‐receptors are dispensable, at least for *WOX4* expression regulation. Intriguingly, another receptor kinase, ERECTA, which interacts with PXY (see section VII), forms differing signalling complexes based on the presence of co‐receptors (Lee *et al*., 2012, 2015; Lin *et al*., 2017). A remaining question for future research is whether the events that occur in response to TDIF binding to PXY depend on the presence or absence of different complex components.

## IV. Events that occur upon binding of TDIF by PXY (i): WOX14‐TMO6‐LBD4 feed‐forward loop regulation

With the aim of integrating TDIF‐PXY with other regulators of vascular development, a transcriptional regulatory network was developed using enhanced yeast‐1‐hybrid (Gaudinier *et al*., 2011). This network was generated by screening promoters from PXY signalling components and other regulators of vascular development against a library of vascular cylinder‐expressed transcription factors (Gaudinier *et al*., 2011; Reece‐Hoyes *et al*., 2011; Smit *et al*., 2020). This integration was necessary because PXY and auxin were known to be integrated via the transcription factor, WOX4. Through genetics, WOX4 was demonstrated to modulate cambium cells' sensitivity to auxin by directly regulating auxin signalling components, including AUX/IAA genes (Suer *et al*., 2011). *wox4* mutants exhibit reduced auxin responsiveness, while *WOX4* overexpression enhances it. These findings establish *WOX4*'s dual role in both receiving input from the PXY‐TDIF pathway and output promoting auxin responsiveness (Suer *et al*., 2011). In total, the transcriptional network comprised 690 transcription factor‐promoter interactions.

To validate the network, a novel transcription factor motif was characterised. *WOX4* and *WOX14* act redundantly downstream of PXY signalling (Etchells *et al*., 2013), and a network motif centred on WOX14, which emerged from the network analysis, was described. Here, *WOX14* was found to act in a feed‐forward loop with *TMO6*, a transcription factor known to be regulated by both auxin and cytokinin, and *LBD4*, which is also cytokinin‐regulated (Smet *et al*., 2019; Smit *et al*., 2020). WOX14 bound to the promoters of *TMO6* and *LBD4*, while TMO6 bound the *LBD4* promoter, generating a feed‐forward loop with LBD4 as the output gene. *wox14* mutants showed reduced transcript levels of both *TMO6* and *LBD4* genes, and this reduction was compounded in *wox4 wox14* double mutants. While *LBD4* expression appeared *TMO6*‐independent under normal conditions, in sensitized genetic backgrounds: *pxy pxl1 pxl2 tmo6* and *wox4 wox14 tmo6*, *LBD4* expression was reduced. These results pointed to a network motif characterised by positive regulation, where *WOX14* promotes the expression of both *TMO6* and *LBD4*, and *TMO6* promotes *LBD4* expression. Moreover, both auxin and TDIF treatment induced *WOX14*, *TMO6* and *LBD4* expression, confirming TDIF/PXY activates the expression of all genes in the feedforward loop.

**Fig. 2:**
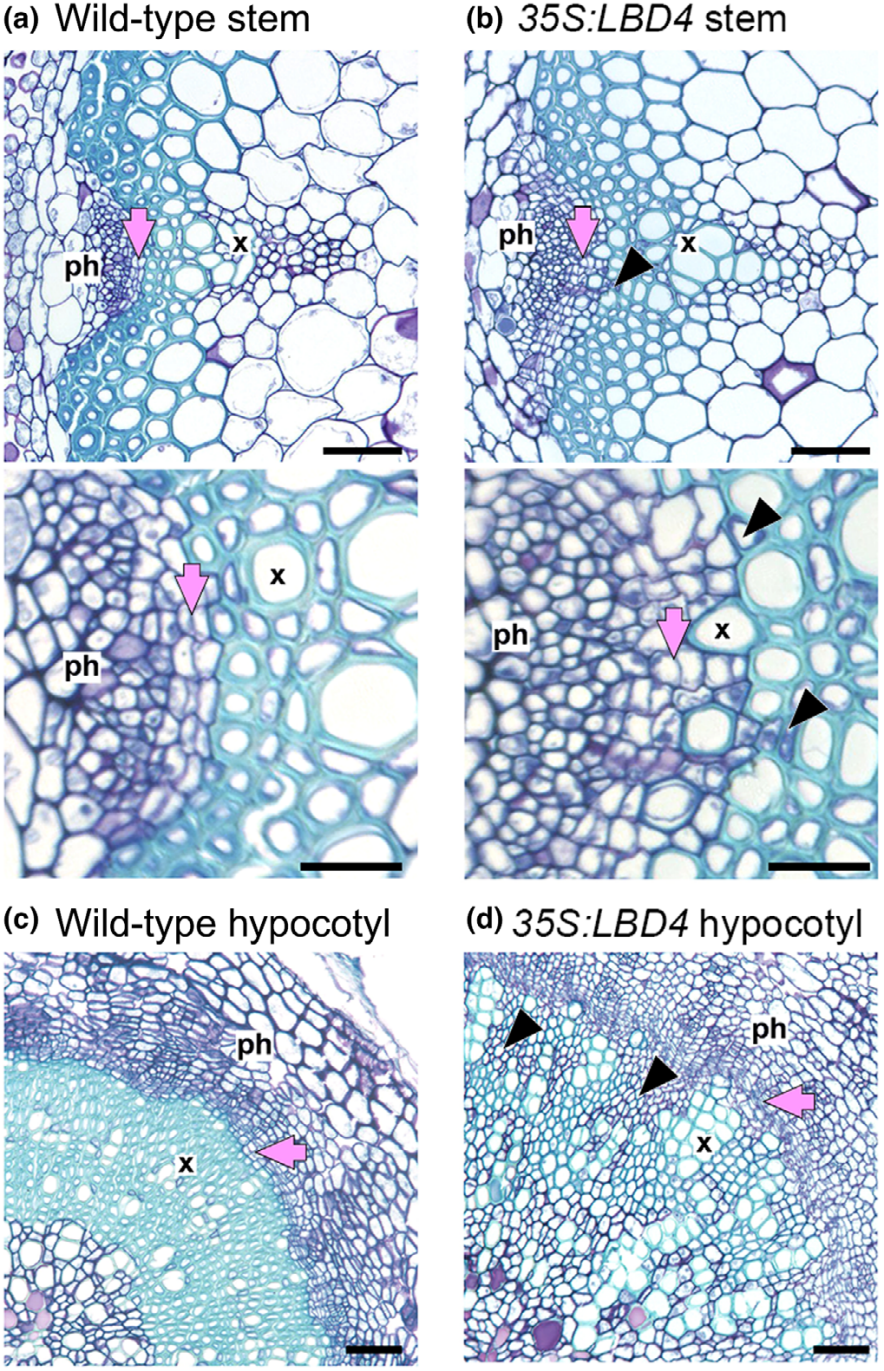
Consequences of overexpression of LBD4, the output gene of the WOX14‐TMO6‐LBD4 feed‐forward loop in *Arabidopsis thaliana*. (a) Vascular bundle from a wild‐type stem showing ordered xylem and phloem, derived from procambium. (b) Differentiation defects in *35S:LBD4* lines with cells in the xylem lacking lignification (black arrowheads). (c) Wild type hypocotyl. (d) *35S:LBD4* hypocotyl showing defects in xylem differentiation (black arrowheads). Bars, upper panels of (a) and (b), and panels (c) and (d) are 50 μM. Bars, lower panels of (a) and (b) are 20 μM. X marks xylem, Ph marks phloem. Pink arrows mark the procambium (a, b) or cambium (c, d).

In the absence of *WOX14*, *TMO6* and *LBD4*, a reduction in cell number was observed in stem vascular bundles in comparison to wild‐type, demonstrating that this feedforward loop is required to promote vascular cell division. Furthermore, genetic analysis with *lbd4* in particular also suggested a role for this network motif in the regulation of vascular organisation, possibly via specification of phloem precursors and repression of xylem differentiation. Here, *LBD4* expression was enriched on the phloem side of procambium in the stem vascular bundle. In TDIF over‐expression lines, ectopic phloem formation was reduced in the absence of *LBD4*. *LBD4* misexpression from a companion cell‐specific promoter resulted in a significant expansion in phloem size, whereas *pxy lbd4* mutants were characterised by significant reductions in phloem expansion. Ectopic expression of *LBD4* in the xylem leads to changes to xylem differentiation (Fig. 2) (Smit *et al*., 2020).

## V. Events that occur upon binding of TDIF by PXY (ii): regulation of MONOPTEROS

Glycogen synthase kinase 3 proteins (GSK3s) represent downstream components of the TDIF‐PXY signalling pathway. PXY and the GSK3, BRASSINOSTEROID INSENSITIVE 2 (BIN2), interact at the plasma membrane. GSK3s BIN2 and BIN2‐LIKE 2 (BIL2) contribute to procambium maintenance by inhibiting xylem cell differentiation via negative regulation of the xylem‐promoting transcription factor BES1 (Kondo *et al*., 2014; Saito *et al*., 2018). Here, the interaction between the PXY kinase domain and BIN2 results in phosphorylation of BES1, a transcription factor that promotes xylem differentiation, thus marking it for degradation.

By contrast, PXY has been reported to repress the activity of a second GSK3 protein, BIN2‐LIKE1 (BIL1). This acts to prevent phosphorylation of MONOPTEROS (MP), an ARF transcriptional regulator. MP phosphorylation by BIL1 suppresses cambial activity by activating ARR7/15 (A‐type ARR transcription factors 7/15), which are negative regulators of cytokinin signalling, with cytokinin being a promoter of cambium activity. PXY was demonstrated to be sufficient to inhibit BIL1‐mediated MP phosphorylation, thus preventing MP phosphorylation and, in turn, reducing *ARR7* and *ARR15* expression (Han *et al*., 2018).

The function of MP in cambium regulation extends beyond regulation of ARR7 and ARR15. An auxin maximum is present in developing xylem cells, which is interpreted by *MP* (and *ARF7* and *ARF19*) (Smetana *et al*., 2019). MP is active under high auxin concentrations, and induction of a dominant active version of MP (MPΔ) was found to promote the expression of *HD‐ZIP III* genes in the xylem. However, MPΔ induction also resulted in elevated *PXY* expression in the xylem and cambium. These observations led to a model for cambium organisation. Specifically, an auxin maximum in xylem precursor cells leads to high MP activity. This in turn leads to high *HD‐ZIP III* expression, which is a determinant of xylem cell fate (and thus loss of stem cell fate). However, MP promotion of PXY expression in neighbouring cells also promotes cambium activity (Smetana *et al*., 2019).

Collectively, the papers described here thus show that MP function remains somewhat enigmatic. On one hand, phosphorylated MP promotes cambium reduction via increased ARR7/15 activity and promotes differentiation via promotion of *HD‐ZIP III* expression. Indeed, mild *mp* mutants demonstrated increases in vascular cambium size and radial growth supporting the idea that MP promotes differentiation. On the other hand, dephosphorylated MP (as a result of PXY signalling) results in lower ARR7/15 activity; MP promotes PXY expression in xylem‐adjacent cambium (Mattsson *et al*., 2003; Vera‐Sirera *et al*., 2015; Brackmann *et al*., 2018; Smetana *et al*., 2019).

A mathematical model that tested positive and negative regulation of cambium activity by MP (which was modelled as a feedback loop) found that patterning was maintained across a higher number of parameter sets when both cambium‐activating and cambium‐repressing reactions were included (Bagdassarian *et al*., 2023). Indeed, the negative feedback loop became essential as MP became more stable (tested in the model by varying the MP degradation rate). Although this requires testing *in planta*, clearly, MP is more stable at higher auxin levels (Bagdassarian *et al*., 2023).

## VI. Events that occur upon binding of TDIF by PXY (iii): regulation of AIL expression to define the cambium stem cells

**Fig. 3:**
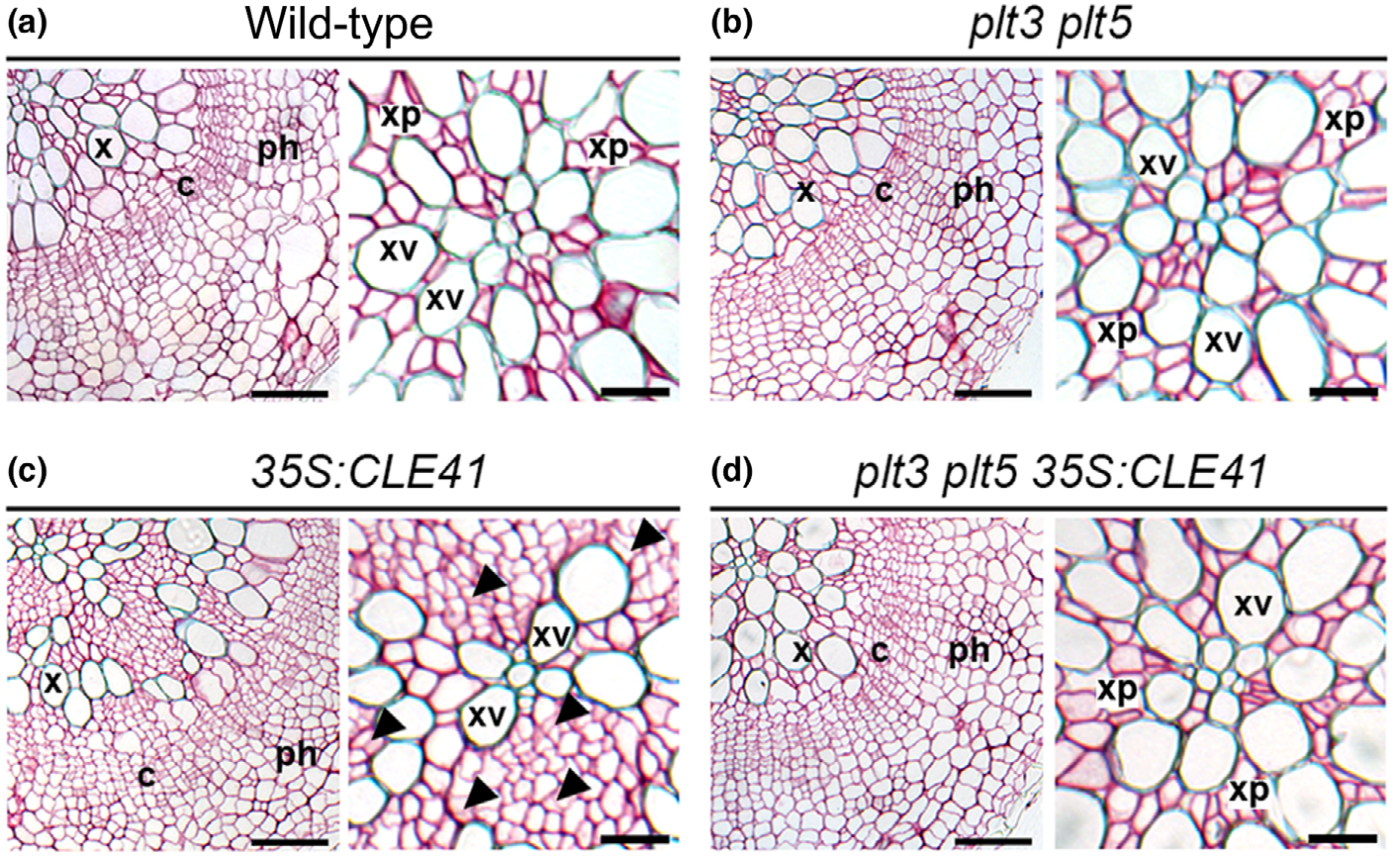
CAIL genes are required for phenotypes associated with constitutive PHLOEM INTERCALATED WITH XYLEM (PXY) activation in *Arabidopsis thaliana*. Transverse sections of hypocotyls from wild‐type (a) and *plt plt5* double mutants (b), with cambium constrained to a discrete zone between xylem and phloem. *35S:CLE41* lines (c) have ectopic PXY activation resulting in cell division within the xylem. *35S:CLE41 plt3 plt5* lines show that CAIL genes are required for *35S:CLE41* phenotypes. Right‐hand panels show hypocotyl quarters; those on the left show a close‐up of the xylem. X is xylem, c is cambium, Ph is phloem, xp are xylem parenchyma and xv are xylem vessels. Black arrowheads mark ectopic cambial cell divisions. Bars, 50 or 20 μM on left and right panels, respectively.

Recently, another robust patterning mechanism was revealed by combining experimental and modelling approaches. The idea of an auxin maximum in the xylem, which activates MP, which in turn activates PXY expression, points to a distribution gradient of PXY that is high on the xylem side of the cambium and depletes towards the phloem (Smetana *et al*., 2019). By contrast, TDIF is expressed in the phloem and forms a gradient, diffusing across the cambium (Hirakawa *et al*., 2008; Etchells & Turner, 2010). This suggested that the balance between the auxin/PXY gradient and the TDIF gradient determines the localization of the cambial stem cells as well as cambium size. The challenge here was that no cambium stem cell factors had been identified to determine cambium stem cell location. However, AINTEGUMENTA (ANT) and three further ANT‐like transcription factors, *PLT3*, *PLT5* and *PLT7* (referred to collectively as Cambium‐expressed AILs; CAILs), were identified as differentially expressed in lines in which TDIF‐PXY signalling had been altered. Further experiments demonstrated that *CAIL* expression is promoted by PXY and that they are required for TDIF overexpression phenotypes (Fig. 3). CAILs are specifically expressed in cambium, and mutation of all four CAILs resulted in loss of cambium identity and consequently secondary growth. Overexpression of CAIL genes resulted in ectopic cambium formation. CAIL expression occurred only in a narrow stem cell domain (one or two cell thickness) within the cambium, and these observations support the idea that the CAIL genes constitute the cambium stem cell factors (Randall *et al*., 2015; Eswaran *et al*., 2024).

The constrained CAIL expression domain contrasts with that of PXY, which is strongest in the xylem domain and tapers off toward the stem cells, suggesting a mechanism is required to constrain CAIL expression to the narrow stem cell domain. To determine the mechanism for defining cambium stem cell positioning, two possibilities were modelled. The first involved network architecture providing a patterning constraint (Eswaran *et al*., 2024). Here, HD‐ZIP III transcription factors, which define xylem identity downstream of auxin, were proposed to mediate repression of *ANT* expression. As the auxin maximum peaks in the xylem and tapers through the cambium, HD‐ZIP III transcription factors repress ANT expression, thereby suppressing expression of the stem cell marker *ANT* and consequently restricting stem cell identity along a phloem‐to‐xylem activity gradient. The second patterning mechanism, which was found to be dominant, involved TDIF sequestration by PXY. Here, an excess of PXY receptor binds and traps TDIF ligand, preventing its egress into the xylem. This creates a localized concentration of active TDIF‐PXY complexes, thus defining the stem cell niche. Evidence for the dominance of TDIF sequestration as the dominant patterning constraint included TDIF overexpression, which expanded ANT expression xylem‐ward. More direct evidence of sequestration of TDIF by PXY came from expression of truncated and kinase‐dead PXY in the phloem. Here, asymmetric PXY‐TDIF turnover in phloem‐side daughters prevented TDIF from reaching the cambium and resulted in a *pxy‐*like phenotype, where cambium stem cells were lost. The sequestration mechanism prevails due to its precise spatial control (1–2 cell layers) and independence from PXY receptor activation, revealing a non‐canonical ‘ligand sink’ function pivotal for stem cell niche patterning (Eswaran *et al*., 2024). The mechanism revealed how the auxin‐promoted organizer‐cell performs cambial stem cell patterning by inducing high levels of PXY in the xylem and cambium, which sequesters TDIF ligands on the edge of the PXY gradient to confine CAIL expression to a discrete domain. This demonstrates that plants are able to utilize opposing morphogen gradients fine‐tuned by sequestration‐based feedback mechanisms to precisely regulate cell positioning and fate decisions. In terms of TDIF‐PXY signalling, an important question for further research is how this mechanism is tuned for plants with different‐sized cambia and differing cambium activities throughout the growing season.

## VII. Parallel pathways to TDIF‐PXY (i): the ERECTA family

There is a striking difference in phenotypic strength of *pxy* mutants and those of its CAIL transcription factor targets. While *pxy* lines undergo disrupted secondary growth (Fisher & Turner, 2007), it is abolished in *plt plt5 plt7 IGE‐ANT* plants (where IGE refers to induced genome editing) (Eswaran *et al*., 2024). Furthermore, *pxy* mutants are enhanced by mutations in their downstream targets (Etchells *et al*., 2013; Kondo *et al*., 2014). This points to mechanisms acting in parallel with PXY signalling. The LRR receptor‐like kinase *ERECTA* (*ER*) is one such factor. *ER* has two paralogues, *ERL1* and *ERL2* (collectively *ER*f for *ERECTA* family). The ligands for ERf receptors belong to the EPIDERMAL PATTERNING FACTOR (EPF) and EPFL‐LIKE (EPFL) peptide family. These peptides are encoded by 11 genes in Arabidopsis and vary in length from 45 to 75 amino acids (Kondo *et al*., 2010; Sugano *et al*., 2010; Takata *et al*., 2013). The ER signalling pathway regulates plant growth and development in multiple settings. *ER* and its homologues collectively regulate stomatal patterning via specification of stomatal stem cell fate and the differentiation of guard cells (Shpak *et al*., 2004). Another LRR receptor‐like protein TMM (TOO MANY MOUTHS) forms complexes with ERfs to perceive ligands EPF1 and EPF2. Moreover, the ERf regulates longitudinal plant growth, with ER promoting organ elongation over a substantially large fraction of the growth period without altering the total growth duration (Shpak *et al*., 2004; Bundy *et al*., 2012; Chen *et al*., 2013). ER also regulates inflorescence architecture (Cai *et al*., 2017); shoot apical meristem function (Uchida *et al*., 2013) and the initiation of leaf primordia (Tameshige *et al*., 2016). Additionally, ER contributes to plant immunity through its interaction with another receptor‐like kinase *BEK1* (Jorda *et al*., 2016; Cai *et al*., 2021).

ER is also an essential regulator of secondary development. ER receptors regulate cell division and xylem fibre formation during secondary growth while repressing xylem expansion in hypocotyls (Ragni *et al*., 2011; Uchida *et al*., 2012; Ikematsu *et al*., 2017; Milhinhos *et al*., 2019). Furthermore, ER physically interacts with SOBIR1 (SUPPRESSOR OF BIR‐1), an LRR‐RK also known as EVERSHED (EVR), which was identified as a regulator of secondary growth using a genome‐wide association study. Xylem differentiation defects caused in *sobir1/evr* mutants were exacerbated by *er* mutations, indicating that *SOBIR1/EVR* and *ERECTA* function together to prevent the precocious xylem fiber development (Milhinhos *et al*., 2019). *ER* and *ERL1* were also shown to redundantly suppress excessive radial growth of the hypocotyl vasculature during secondary growth in addition to preventing premature initiation of the fiber differentiation process in the hypocotyl xylem (Ikematsu *et al*., 2017). Repression of xylem differentiation is a critical component of cambium homeostasis.

*ER*, *ERL1* and *ERL2* are expressed in most hypocotyl cell types, including the cambium, xylem initials and periderm, although *ERL2* promoter activity is only evident in mature hypocotyls (Ikematsu *et al*., 2017; Wang *et al*., 2019). Its cognate ligands, *CHALLAH‐LIKE 2*/*EPIDERMAL PATTERNING FACTOR‐LIKE 4* (*CLL2*/*EPFL4*) and *CHALLAH* (*CHAL*/*EPFL6*) are also expressed broadly, although expression appears greatest in the xylem parenchyma and differentiating xylem (Wang *et al*., 2019).

**Fig. 4:**
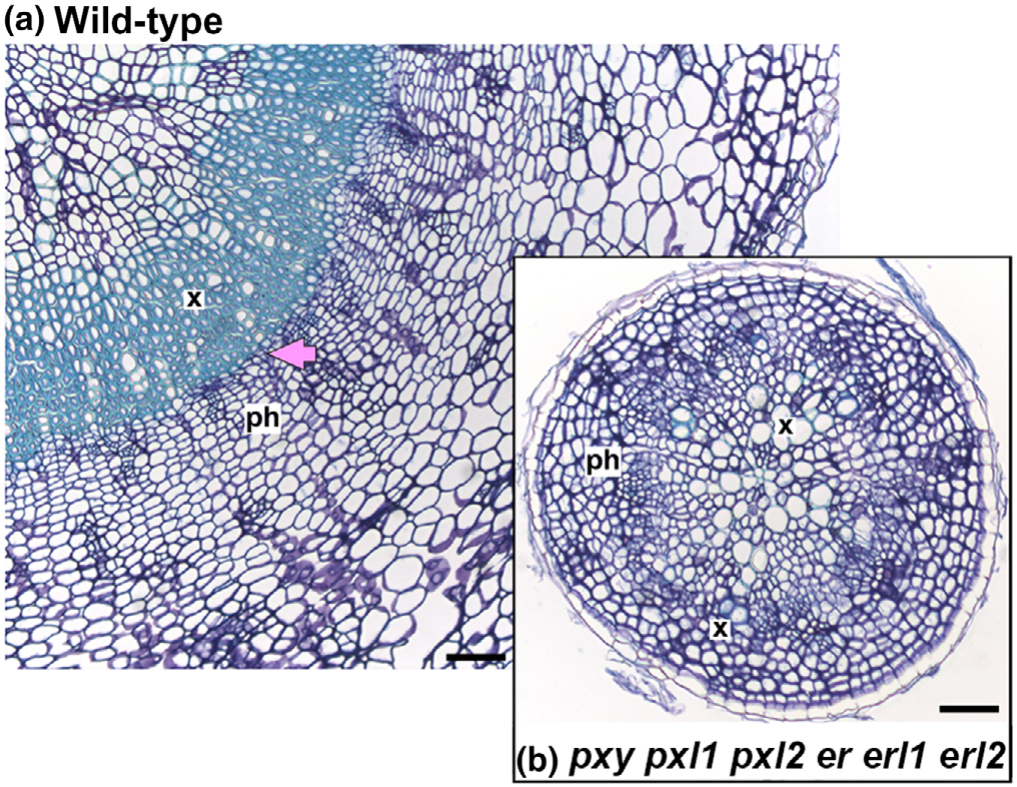
Comparison of wild‐type and *pxy pxl1 pxl2 er erl1 erl2 Arabidopsis thaliana* plants. Transverse sections of hypocotyls from wild‐type (a) and *pxy pxl1 pxl2 er erl1 elr2* (b) lines at 28 d, showing dramatic differences in growth and tissue organization. Bars, 50 μM. X marks xylem; Ph marks phloem. Pink arrow marks the cambium.

Crosstalk between TDIF‐PXY, PXL1, PXL2 and EPFL4/6‐ERf has been described, with PXY and ER families interacting to control vascular cell proliferation, cell size and organisation (Etchells *et al*., 2013; Uchida *et al*., 2013; Wang *et al*., 2019). *pxy* mutants demonstrate a change to vascular bundle shape, being narrower on the radial axis but wider on the tangential. Xylem and phloem also become intercalated as the vascular meristem is perturbed (Fisher & Turner, 2007). These phenotypes were enhanced by *er* (Etchells *et al*., 2013). Furthermore, *pxy pxl1 pxl2 er* lines exhibited fewer cells in stem vascular bundles compared to either *pxy er* or *px*y lines, showing that *pxl1* and *pxl2* genetically interact with *er. Complete loss of both PXY and ER families results* in severe vascular defects in both stems and hypocotyls. In the stem, *pxy pxl1 pxl2 er erl1 erl2* sextuple mutants showed dramatic vascular abnormalities, including dramatic reductions in cell number, smaller xylem vessels, intercalated xylem and phloem, and missing phloem‐cap resulting in severe organisation defects. In hypocotyls, the sextuple mutant is characterised by dramatic reductions in secondary growth, such that the cambium does not form a complete ring (Fig. 4) (Wang *et al*., 2019).

This failure to fully transition to secondary growth represents the clearest evidence of PXY and ER families acting together to coordinate vascular development. While both *pxy pxl1 pxl2* and *er erl1 erl2* triple mutants demonstrate a delay to the onset of cambium formation, they nevertheless still make the transition (Wang *et al*., 2019). The nature of the interaction has been a matter of debate. Clearly, the genetic interaction is not that of epistasis, which would be expected if PXY and ER families constituted components of the same linear genetic pathway. Given the pleiotropic nature of ER, especially its role in regulating xylem differentiation, this lack of epistasis is not unexpected. However, the phenotypes are not additive. For example, *er erl1* doubles have been described with an increase in radial growth (Ikematsu *et al*., 2017), yet *pxy pxl1 pxl2 er* lines show a reduction. Rather, the enhancement of *pxy* by *er* demonstrates that there is coordination between these families. The genetic interactions between PXf (PXY, PXL1 and PXL2) and ERf may be influenced by various molecular‐level factors, including at the transcriptional level, shared downstream phospho‐targets and/or protein complex formation (Mani *et al*., 2008). Recently, two preprint publications have shed light on the nature of the interaction. In the first, a constitutively active version of ER expressed under the control of the PXY promoter was found to demonstrate dramatic increases in cambium formation, which confirms that ER activity can promote cambium activity in a similar manner to PXY. Furthermore, ANT, a CAIL transcriptional target of TDIF‐PXY signalling was found to be a target of ER signalling in the cambium (Xiao *et al*., 2025). The second found that ectopic cambium formation caused by constitutive TDIF production (*IRX3:CLE41*) was partially attenuated by loss of *er*, and that *ANT* levels were lower in *IRX3:CLE41 er* lines than in *IRX3:CLE41* alone. Here, members of the PXY family were also found to form complexes with members of the ER family. This leads to a model whereby PXY and ER family members form complexes at the plasma membrane to coordinate signalling in the regulation of downstream targets (He *et al*., 2024).

## VIII. Parallel pathways to TDIF‐PXY (ii): ethylene

The gaseous hormone ethylene plays a significant role in cambial growth by regulating ERF transcription factors, of which a subset is also influenced by jasmonic acid (JA) and PXY signalling (Pauwels *et al*., 2008; Etchells *et al*., 2012; Cai *et al*., 2014). Ethylene promotes radial growth and vascular cell division, as evidenced by studies on tension wood formation, ethylene‐exposed tree stems and ethylene‐overproducing mutants in Arabidopsis (Love *et al*., 2009; Vahala *et al*., 2013). *ERF* genes, such as *ERF1*, *ERF11*, *ERF018* and *ERF109*, are upregulated in *pxy* mutants. Given that ethylene is a promoter of cell division in the cambium, this suggested a level of coordination between PXY and ethylene signalling, and that in the absence of PXY, ethylene‐mediated promotion of cambium activity was enhanced. As such, ethylene was proposed to act in a compensatory manner to PXY. *pxy ein2* mutants supported this hypothesis as they demonstrated greater reductions in cambium activity than *pxy* single mutants (Alonso *et al*., 1999; Sehr *et al*., 2010; Etchells *et al*., 2012).

ACS (1‐aminocyclopropane‐1‐carboxylic acid (ACC) synthase) is considered to be the rate‐limiting enzyme in ethylene biosynthesis (Bleecker & Kende, 2000). In Arabidopsis, a dominant *ACS7* allele, *acs7‐d*, which was generated by activation tagging, exhibited enhanced cambium activity and defective cell wall formation in xylem fibres, alongside increased levels of ethylene production. Furthermore, loss of ACS genes was found to result in reduced cambium development. When *acs7‐d wox4‐1* double mutants were generated, similar phenotypes to *wox4‐1* were observed. Thus, the enhanced cambium activity in plants with elevated ethylene levels was *WOX4* dependent. This epistasis places *WOX4* downstream of the ethylene signalling (S. Yang *et al*., 2020).

The observation that *WOX4* is required for ethylene‐mediated cambium cell division provides context to the relationship between ethylene and TDIF‐PXY signalling by suggesting that these signalling pathways converge at *WOX4*. It had earlier been shown that ERF gene expression was elevated in the *wox4* mutant (Etchells *et al*., 2012), and one explanation for this is the involvement of feedback regulation. Given that *WOX4* is activated by auxin, and a downstream transcriptional target of ethylene and TDIF signalling, *WOX4* appears to act as a convergence point for regulating cambium activity. On the other hand, the *wox4* phenotype in cambium development and stem cell maintenance is weaker than that of several other cambium mutants, and as such, must only provide part of the picture. It is striking that in rose, ethylene promotes cambium cell division via activation of a homeodomain transcription factor, RhPMP1, which in turn activates expression of auxin biosynthesis genes (Yu *et al*., 2023). Furthermore, members of the *ETHYLENE RESPONSE FACTOR* (*ERF*) transcript factor family, *ERF018* and *ERF109*, are upregulated in *pxy* mutants and responsive to Jasmonic acid (JA), and JA itself also promotes vascular cell division (Pauwels *et al*., 2008; Sehr *et al*., 2010; Cai *et al*., 2014). Thus, it would be interesting to determine the level of dependency of JA activity upon WOX4.

## IX. Parallel pathways to TDIF‐PXY (iii): cytokinin

Cytokinin is a crucial regulator of vascular development. iP‐type cytokinin is one of the biologically important cytokinins, and the first steps of its biosynthesis are catalyzed by ATP/ADP isopentenyl transferases (ATP/ADP IPTs) (Kakimoto, 2001). A quadruple mutant in which four of these genes have been removed, *ipt1 ipt3 ipt5 ipt7*, which has severely reduced cytokinin levels, lacks vascular cambium in the root and exhibits a significant reduction in the number and size of vascular bundles in the stem. Exogenous application of cytokinin rescues these defects, and, as such, cytokinins are regulators of cambial activity (Matsumoto‐Kitano *et al*., 2008). Further, *CYTOKININ‐INDEPENDENT 1* (*CKI1*), a histidine kinase, was indicated to function in cambium development by initiating the cytokinin signalling phosphorelay, inducing cytokinin response, and further increasing cambial cell number. *CKI1* expression is observed in vascular tissues of inflorescence stems, and CKI1 forms homodimers both *in vitro* and *in planta* (Hejatko *et al*., 2009). Loss‐of‐function mutants of cytokinin receptors *AHK2* (*ARABIDOPSIS HISTIDINE KINASE2*) and *AHK3* show defects in procambium proliferation and an absence of secondary growth. *CKI1* overexpression partially rescues *ahk2 ahk3* phenotypes in vascular tissue (Mahonen *et al*., 2006; Hejatko *et al*., 2009). Cytokinin activity in the cambium is in part mediated by transcription factors LBD3 and LBD4 (Ye *et al*., 2021).

Evidence suggests that integration points between cytokinin‐mediated control of vascular development and TDIF‐PXY signalling occur upstream and downstream of cytokinin. AHL15 is a transcription factor which acts upstream of cytokinin, as it has been shown to promote the expression of cytokinin biosynthesis genes *IPT3*, *IPT7* and *LOG4*. The TDIF‐PXY pathway is essential for the function AHL15 plays in vascular development  (Rahimi *et al*., 2022). One explanation for this observation is PXY‐mediated regulation of the feedforward loop involving *WOX14*, *TMO6* and *LBD4* (Smit *et al*., 2020). Both *TMO6* and *LBD4* are also cytokinin signalling targets. As such, the increase in cambium activity observed in *PXYpro:AHL15* plants was observed to be suppressed in *pxy wox4 wox14 PXYpro:AHL15* plants (Rahimi *et al*., 2022). Here, although cytokinin biosynthesis is likely elevated via activation of *IPT3*, *IPT7* and *LOG4* by the *PXYpro:AHL15* construct, this may not have caused an increase in cambium activity because with PXY signalling absent, expression of cytokinin targets TMO6 and LBD4 would be attenuated.

## X. Perspectives

In recent years, our understanding of PXY signalling and the complex network involving multiple phytohormones and peptide signalling modules in radial growth has advanced greatly. Key regulators have been found, but in many cases, a mechanistic detail is lacking. One example here is the cambium plasma membrane. XVP, SERKs, ER, GSK3s and PXY itself interact there, but details of what promotes these components to come together, if they are all present in a larger complex, or whether different complexes form with varying components that control different targets via independent signal transduction pathways is a key question. Further questions surround the turnover of such complexes. The determination of these mechanisms represents an exciting prospect.

A second set of questions surrounds transcription factor regulation. The transcriptional response to PXY binding TDIF now encompasses many factors: WOX4, WOX14, PLT3, PLT5, PLT7, ANT, LBD4, TMO6. The best‐characterized transcriptional response mechanisms involve direct regulation of BES1 and MP by GSK3 phosphorylation; but in the case of the AIL and WOX4 transcription factors, the mechanism that triggers the expression change is unknown. How this network collectively generates a robust pattern remains an open question.

Plant growth and development are dynamic processes. In Arabidopsis, it was demonstrated that components of the TDIF‐PXY system are subject to regulation by light in seedlings. Here, in the dark, hyponastic growth is favoured with little need to supply water for photosynthesis. Under such conditions, expression of *PXL1* and *CLE44* was high, activated by PIF transcription factors, thus preventing xylem differentiation. Upon perception of light, PIFs are repressed by photoreceptors; *PXL1* and *CLE44* expression falls, and xylem differentiation proceeds as photosynthesis and cell division are initiated (Ghosh *et al*., 2022). It seems unlikely that this will be the only instance where environmental factors act through TDIF‐PXY. Not least, nearly everyone is familiar with the idea that the space between annual growth rings in the wood of trees represents a readout of the growth conditions in a particular season. The question of how seasonality and other environmental inputs to core developmental networks in the future will determine how plant forms might change as we enter a period of increasing climate uncertainty.

Finally, our knowledge of TDIF‐PXY signalling and the transcription network that it regulates has a degree of depth in a single species, *Arabidopsis thaliana*, with genetic data also in *Marchantia polymorpha* and hybrid poplar for some core components. However, the presence of such TDIF and PXY‐like components within the green plant lineage from bryophytes to angiosperms, incorporating plants with diverse morphologies and life cycles, suggests that TDIF‐PXY function may have been modified for each. It is tempting to speculate that in some cases, modification of TDIF‐PXY may have been a driver of changes in architecture that enabled plants to adapt to different niches.

It has been almost 20 years since the first description of TDIF in 2006, followed by PXY in 2007; and although our understanding of this signalling pathway has significantly improved, there remain as many interesting questions as there are answers.

## Competing interests

None declared.

## Author contributions

QH conceptualized the manuscript with guidance from JPE. QH and AG‐K wrote the manuscript with contributions from JPE and EEM. JPE and EEM compiled the figures.

## Disclaimer

The New Phytologist Foundation remains neutral with regard to jurisdictional claims in maps and in any institutional affiliations.
